# A high triglyceride-glucose index correlates with cognitive impairment in Parkinson’s disease: a cross-sectional study

**DOI:** 10.3389/fnins.2025.1620118

**Published:** 2025-08-28

**Authors:** Yongqing Cheng, Shuangfei You, Xin Wang, Yingchao Ge, Lei Li, Taojie Ren, Songjie Chen, Guojun He, Shouru Xue

**Affiliations:** ^1^Department of Neurology, The Yancheng Clinical College of Xuzhou Medical University, The First People’s Hospital of Yancheng, Yancheng, Jiangsu, China; ^2^Department of Neurology, The First Affiliated Hospital of Soochow University, Suzhou, Jiangsu, China; ^3^Department of Radiology, The Yancheng Clinical College of Xuzhou Medical University, The First People’s Hospital of Yancheng, Yancheng, Jiangsu, China; ^4^Department of Neurology, Qidong Hospital Affiliated to Nantong University, Nantong, Jiangsu, China

**Keywords:** triglyceride-glucose index, insulin resistance, cognitive impairment in Parkinson’s disease, biomarker, predictor

## Abstract

**Background:**

Insulin resistance (IR) is proved be involved in the pathophysiology of Parkinson’s disease (PD). As an effective surrogate marker of IR, the correlation between the triglyceride-glucose (TyG) index and PD remains unclear. This cross-sectional study aimed to explore the association between the TyG index and cognitive impairment in PD (PDCI).

**Methods:**

Patients with sporadic PD were consecutively enrolled between May 2022 and October 2023. The cognitive function was evaluated using the Montreal Cognitive Assessment (MoCA). The Spearman correlation analysis was used to evaluate the correlation between TyG index and MoCA score, Unified-Parkinson Disease Rating Scale (UPDRS) III and peripheral blood oxidative stress markers, respectively. Logistic regression analysis was performed to explore the correlation between TyG and PDCI and dementia in PD (PDD).

**Results:**

A total of 78 patients were enrolled, of whom 50 (64.1%) were diagnosed with PDCI [26 with mild cognitive impairment (MCI) and 24 with PDD]. The TyG index in patients with dementia and MCI were higher than those with normal cognition (9.32 ± 0.43 vs. 8.90 ± 0.47 vs. 8.51 ± 0.46, *P* < 0.001). The Spearman correlation analysis demonstrated that TyG was negatively correlated with MoCA (*r* = −0.704, *P* < 0.001) and superoxide dismutase (*r* = −0.244, *P* = 0.031), but positively correlated with UPDRS III (*r* = 0.246, *P* = 0.030). Multivariate logistic regression analysis showed that TyG was independently associated with PDCI regardless of whether it was used as a continuous variable (OR = 6.177, 95% CI = 1.590–24.000) or a tertile variable (OR = 5.478, 95% CI = 1.030–29.132). This association persisted after excluding patients with diabetes. The receiver operating characteristic (ROC) analysis suggested that the area under the curve (AUC) of TyG for predicting PDCI was 0.805 (95% CI = 0.707–0.903, *P* < 0.001).

**Conclusion:**

Elevated TyG levels were associated with an increased likelihood of PDCI in patients with PD.

## 1 Introduction

Parkinson’s disease is the second most common neurodegenerative disease, and its incidence is increasing. Cognitive dysfunction is one of the most common non-motor symptoms, which has attracted extending attention (Hussein et al., 2023; Aarsland et al., 2017). Type 2 diabetes mellitus (T2DM) is also a prevalent disease in the elderly. It has been reported to share a common biological mechanism with neurodegenerative diseases such as PD and Alzheimer’s disease (AD) (Athauda and Foltynie, 2016; Peng et al., 2018). As an important pathogenic factor of T2DM, the correlation between insulin resistance (IR) and neurodegeneration has become a hot research topic in recent years. Accumulating evidence suggests that patients with PD have a higher incidence of IR, which in turn is associated with more severe PD symptoms, faster PD progression, and a higher risk of cognitive impairment in PD (PDCI) (Peng et al., 2018; Butterfield et al., 2022; de Pablo-Fernández et al., 2021).

The triglyceride glucose (TyG) index, an effective surrogate marker of IR, was initially proposed by Simental-Mendía et al. (2008) and Bastard et al. (2012). This index, converted from fasting triglyceride and fasting blood glucose, has been widely used in many fields in recent years due to easy clinical availability and cost-effectiveness. A large number of clinical studies have shown that the TyG index is closely related to cardiovascular diseases, stroke, and dementia (Li et al., 2022; Hong et al., 2021; Wang et al., 2022). However, there have been few reports on the correlation between TyG and PD. Therefore, we designed this cross-sectional study to investigate the association of the TyG index with cognitive impairment and oxidative stress markers in PD patients.

## 2 Materials and methods

This cross-sectional study was conducted from May 2022 to October 2023 in the First People’s Hospital of Yancheng, a tertiary teaching hospital. All subjects were recruited from the inpatient department of the hospital, and all patients provided written informed consent. The study was approved by the Yancheng First People’s Hospital Medical Ethics Committee and followed the Declaration of Helsinki.

### 2.1 Participants

The inclusion criteria were as follows: (1) age ranged from 50 to 85 years, (2) diagnosed as sporadic PD according to the Movement Disorder Society Clinical Diagnostic Criteria, (3) capable of completing various questionnaires and assessments. The exclusion criteria included: (1) Combined with other diseases that may cause cognitive impairment, such as AD, vascular cognitive impairment (VCD), frontotemporal dementia (FTD), stroke, brain tumors, etc. (2) Severe depression and other psychiatric disorders, (3) with hearing or speech disabilities that may affect the scale evaluation, (4) use of nootropics, antilipemic, antipsychotic drugs within 3 months.

### 2.2 Clinical and laboratory data

All participants received a standard questionnaire and clinical assessment performed by trained neurologists after admission. The baseline demographic data, including age, gender, education level, duration and subtypes of PD, and past medical history, including hypertension and diabetes, were collected. Education level was graded into three levels according to the years of education: illiterate (education years < 1), primary school (1 ≤ education years ≤ 6), and secondary school or above (education years > 6). Clinical assessments were performed during the “on” state of medication, with modified Hoehn and Yahr stage (H&Y) for the severity of PD and subscale III of the Unified Parkinson’s Disease Rating Scale (UPDRS III) for the severity of PD motor disorder. The overall cognition was evaluated using the Montreal Cognitive Assessment (MoCA), and the function of the cognitive subdomains was assessed using the subitems in MoCA. We add one point to the total MoCA scores for those with less than 12 years of education.

Laboratory data included triglycerides (TG), fasting plasma glucose (FPG), and peripheral blood oxidative stress markers, including uric acid, glutathione (GSH), and superoxide dismutase (SOD). Fasting blood samples were collected from all patients the following day of admission. TG, FPG, and uric acid were tested in the laboratory department of our hospital by a laboratory physician using an automatic biochemical analyzer. While GSH and SOD were tested using commercially available ELISA kits. The TyG Index was calculated as Ln [fasting TG (mg/dL) × FPG (mg/dL)/2] (Bastard et al., 2012).

### 2.3 Definition of cognitive function

All subjects were divided into two subgroups according to cognitive function: PD with normal cognition (PDNC) and PD with cognitive impairment (PDCI). PDCI was divided into mild cognitive impairment (PDMCI) and dementia (PDD) according to the severity of cognitive impairment. PD-MCI was diagnosed based on Level I criteria recommended by the Movement Disorder Society. 11 Patients with MoCA ≥ 26 were classified into the PDNC subgroup; MoCA scores < 26 and ≥21 were defined as PDMCI (Litvan et al., 2012). PDD was defined as a MoCA score < 21 with impaired activities of daily living (Becker et al., 2021).

### 2.4 Statistics

Statistical analyses were performed using SPSS version 23.0 (IBM SPSS Inc.) and GraphPad Prism version 9.3.1 (GraphPad Software, San Diego, CA, USA). Continuous variables were presented as the mean (standard deviation), or median (25% and 75% interquartile) according to the distribution characteristics. Categorical variables were presented as numbers (percentages). Differences in baseline characteristics between TyG tertiles or PD cognitive subgroups were analyzed by variance analysis, the Mann-Whitney or Kruskal-Wallis test for continuous variables and the chi-square test for categorical variables. Spearman correlation analysis was applied to analyze the correlation between TyG and other continuous variables, including MoCA score, UPDRS III, GSH and SOD. Logistic regression analysis was performed with TyG as a continuous variable and a tertile variable to verify the correlation between TyG and PDCI or PDD, respectively. Univariable binomial logistic regression analysis was conducted to screen the potentially relevant variables. We then performed multivariable regression analysis adjusting for gender, age (Model 2), and gender, age + other covariates with *P* < 0.1 screened by univariate regression (Model 3). Due to the low prevalence of diabetes (*n* = 9) and its balanced distribution across TyG/PDCI groups, diabetic status was not included as a covariate in primary models. Stratified analyses were conducted to assess the TyG–PDCI association in non-diabetic patients. The corresponding odds ratios (ORs) and 95% CIs were calculated. Furthermore, we conducted receiver operating characteristic (ROC) analysis to evaluate the predictive ability of TyG to PDCI. A value of *P* < 0.05 was considered to be statistically significant.

## 3 Results

### 3.1 Baseline characteristics among TyG tertiles

**FIGURE 1:**
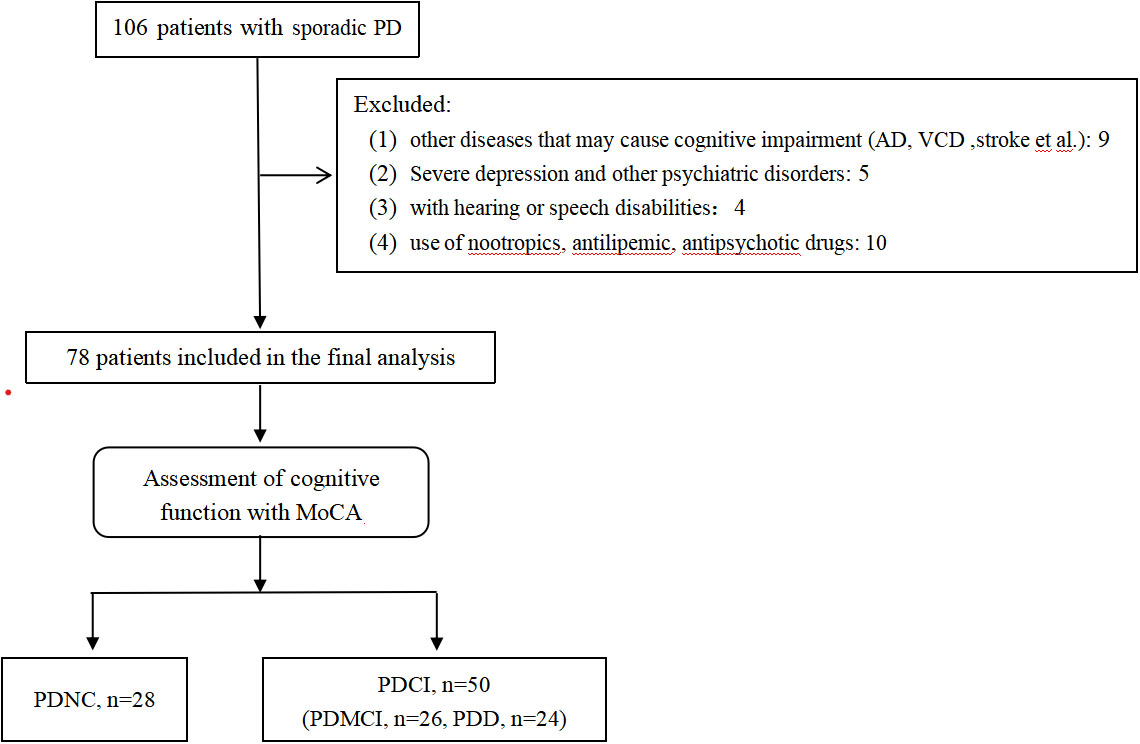
Flowchart of the study.

A total of 106 PD patients (13 cases with diabetes) were consecutively screened into the study. Based on the exclusion criteria, 28 patients were excluded (including 4 cases with diabetes). Seventy-eight patients were finally included in the study, with a median age of 71 (IQR, 65–75.3) and 47 (60.3%) males (Figure 1). All patients were divided into groups based on tertiles of TyG as follows: Tertile 1, ≤8.64; Tertile 2, 8.65–9.14; Tertile 3, ≥9.15. Differences in baseline characteristics of the patients within the three subgroups are shown in Table 1. There were significant statistical differences among the three subgroups in the following variables: age (*p* = 0.001), gender (*p* = 0.031), education (*p* = 0.01), MoCA score (*p* < 0.001) and cognitive function (*p* < 0.001).

**TABLE 1:** Baseline characteristics among patients according to tertiles of TyG.

Baseline characteristic	Tertile 1 (*n* = 27)	Tertile 2 (*n* = 25)	Tertile 3 (*n* = 26)	*P*
**Demographics**				
Age, median (IQR) (years)	66.0 (60.0, 70.0)	74.0 (68.0, 77.5)^a^	74.0 (69.0, 77.5)^a^	0.001
Male, *n* (%)	21 (77.8)	15 (60.0)^a^	11 (42.3)^b^	0.031
Education, *n* (%)				0.010
Illiteracy	4 (14.8)	7 (28.0)	10 (38.5)	
Primary	3 (11.1)	10 (40.0)^a^	7 (26.9)^a^^b^	
Secondary	20 (74.1)	8 (32.0)^a^	9 (34.6)^a^	
**Medical history**				
Hypertention, *n* (%)	5 (18.5)	7 (28.0)	9 (34.6)	0.414
Diabetes mellitus, *n* (%)	2 (7.4)	2 (8.0)	5 (19.2)	0.322
**Clinical characteristics**				
Disease duration, median (IQR) (yeas)	3.0 (2.0, 4.0)	3.5 (2.0, 5.0)	4.8 (2.4, 6.3)	0.314
Tremor predominated subtype, *n* (%)	11 (40.7)	6 (25.0)	10 (38.5)	0.454
H&Y stage, median (IQR)	2.0 (1.5, 2.5)	2.5 (2.0, 3.0)	2.5 (1.9, 3.3)	0.028
UPDRS III, median (IQR)	21 (14.0, 36.0)	25 (18.0, 46.0)	24 (19.5, 58.8)	0.163
MoCA, median (IQR)	28 (25, 29)	25 (21.5, 27)^a^	20.5 (15.5, 24)^a^^b^	<0.001
Cognitive function				<0.001
PDNC, *n* (%)	17 (63.0)	8 (32.0)^a^	3 (11.5)^a^^b^	
PDMCI, *n* (%)	8 (29.6)	10 (40.0)	8 (30.8)	
PDD, *n* (%)	2 (7.4)	7 (28)^a^	15 (57.7)^a^^b^	
**Laboratory characteristics**				
TG, median (IQR) (mmol/L)	1.18 (0.91, 1.34)	1.87 (1.75, 2.01)	2.70 (2.27, 3.19)	<0.001
FPG, median (IQR) (mmol/L)	4.55 (4.27, 5.08)	5.58 (5.14, 5.81)	5.73 (5.43, 5.98)	<0.001
Ferroprotein, median (IQR) (ng/ml)	138.12 (97.30, 294.97)	91.26 (42.60, 152.96)	158.44 (79.01, 207.81)	0.075
Uric acid, median (IQR) (umol/l)	295.20 (240.20, 339.50)	264.9 (212.25, 337.10)	283.65 (210.05, 321.28)	0.530
GSH, mean ± SD (umol/l)	27.73 ± 9.74	22.63 ± 8.08	24.19 ± 9.21	0.201
SOD, mean ± SD (U/ml)	67.11 ± 27.91	60.33 ± 29.31	52.24 ± 27.60	0.224

a, *P* < 0.05 compared to Tertile 1; b, *P* < 0.05 compared to Tertile 2. Medians and IQRs summarize central tendency/spread; individual values (including extremes beyond IQRs) are shown in Figures 2, 3. TyG, triglyceride-glucose; IQR, interquartile range; H&Y, Hoehn and Yahr; UPDRS III, subscale III of the Unified Parkinson’s Disease Rating Scale; MoCA, Montreal Cognitive Assessment; PDNC, Parkinson’s disease with normal cognition; PDMCI, mild cognitive impairment in Parkinson’s disease; PDD, dementia in Parkinson’s disease; TG, triglyceride; FPG, Fasting plasma glucose; GSH, glutathione; SD, standard deviation; SOD, superoxide dismutase.

### 3.2 Comparison of baseline characteristics among cognitive subgroups

All patients were divided into PDNC, PCMCI, and PDD subgroups according to their cognitive function, and the baseline characteristics of the patients in each subset were exhibited in Table 2. Among all 78 patients, 50 patients were diagnosed with cognitive impairment (PDCI), including 26 patients with PDMCI and 24 patients with PDD. Compared with the PDNC subgroup, the PDCI subgroup had a higher level of age, disease duration, H&Y grade, and TyG, a lower level of education, and a lower proportion of males. However, in the comparison of non-PDD and PDD subgroups, in addition to the above variables, there were also significant differences in the levels of oxidative stress markers GSH and SOD between the two groups (Table 2).

**TABLE 2:** Comparison of baseline characteristics among PDNC, PDCI and PDD subgroups.

Baseline characteristic	PDNC (*n* = 28)	PDCI (*n* = 50)		*P1*	*P2*
		PDMCI (*n* = 26)	PDD (*n* = 24)		
**Demographics**					
Age, (IQR) (years)	66.5 (61.5, 70.5)	73.5 (64.8, 77.0)	75.0 (70.5, 79.8)	<0.001	0.001
Male, *n* (%)	23 (82.1)	16 (61.5)	8 (33.3)	0.003	0.001
Education, *n* (%)				0.025	0.005
Illiteracy	5 (17.9)	5 (19.2)	11 (45.8)		
Primary	4 (14.3)	8 (30.8)	8 (33.3)		
Secondary	19 (67.8)	13 (50.0)	5 (20.8)		
**Medical history**					
Hypertention, *n* (%)	8 (28.6.3)	7 (26.9)	9 (37.5)	0.753	0.391
Diebetes, *n* (%)	3 (10.7)	2 (7.7)	4 (16.7)	0.865	0.345
**Clinical characteristics**					
Duration, median (IQR) (years)	3.0 (2.0, 4.0)	3.0 (2.0, 4.1)	5.3 (3.1, 7.8)	0.026	<0.001
Tremor predominanted, *n* (%)	10 (35.7)	7 (28.0)	10 (41.7)	0.928	0.414
H&Y stage, median (IQR) (years)	2 (1.5, 2.5)	2 (1.5, 3.0)	3.0 (2.1, 4.0)	0.014	0.003
UPDRS III, median (IQR)	24.5 (15.3, 31.5)	20 (13.8, 43.8)	35 (20.0, 56.5)	0.136	0.012
MoCA, median (IQR)	28 (27, 29)	25 (24, 25)^a^	18 (14.3, 19.8)	<0.001	<0.001
**Laboratory characteristics**					
TG, median (IQR) (mmol/L)	1.30 (1.06, 1.84)	1.80 (1.27, 2.16)	2.38 (1.93, 3.13)	<0.001	<0.001
FPG, median (IQR) (mmol/L)	4.57 (4.26, 5.14)	5.38 (4.88, 5.67)	5.69 (5.15, 5.89)	<0.001	0.001
TyG index, median (IQR) (%)	8.51 ± 0.46	8.90 ± 0.47	9.32 ± 0.43	<0.001	<0.001
Ferroprotein, median (IQR) (ng/ml)	138.54 (96.58, 300.54)	119.56 (64.49, 193.83)	115.33 (57.81, 174.35)	0.193	0.247
Uric acid, median (IQR) (umol/l)	285.40 (219.18, 348.13)	291.80 (242.15, 337.68)	264.00 (208.20, 318.38)	0.376	0.163
GSH, mean ± SD (umol/l)	26.34 ± 7.45	26.89 ± 10.30	21.11 ± 8.98	0.333	0.024
SOD, mean ± SD (U/ml)	67.91 (35.02, 80.30)	73.08 (44.10, 84.74)	41.94 (30.55, 58.38)	0.346	0.009

P1, Comparison between PDNC and PDCI; P2, Comparison between non-PDD and PDD. Medians and IQRs summarize central tendency/spread; individual values (including extremes beyond IQRs) are shown in Figures 2, 3. TyG, triglyceride-glucose; IQR, interquartile range; LEDD, levodopa equivalent daily dose; H&Y, Hoehn and Yahr; UPDRS III, subscale III of the Unified Parkinson’s Disease Rating Scale; MoCA, Montreal Cognitive Assessment; PDNC, Parkinson’s disease with normal cognition; PDMCI, mild cognitive impairment in Parkinson’s disease; PDD, dementia in Parkinson’s disease; TG, triglyceride; FPG, Fasting plasma glucose; Fe, ferrum; GSH, glutathione; SD, standard deviation; SOD, superoxide dismutase.

### 3.3 Correlation analysis of the TyG index with MoCA score and other PD-related variables

Correlation analysis of the TyG index with MoCA, UPDRS III, and peripheral blood markers of oxidative stress was performed using Spearman correlation analysis. According to the results shown in Figures 2, 3, TyG was negatively correlated with MoCA score (*r* = −0.704, *P* < 0.001) and positively correlated with UPDRS III (*r* = 0.246, *P* = 0.030) (Figure 2). When it comes to markers of oxidative stress, we found that TyG was negatively correlated with SOD (*r* = −0.244, *P* = 0.031) but not with uric acid (*r* = −0.221, *P* = 0.052) and GSH (*r* = −0.197, *P* = 0.083) (Figure 3).

**FIGURE 2:**
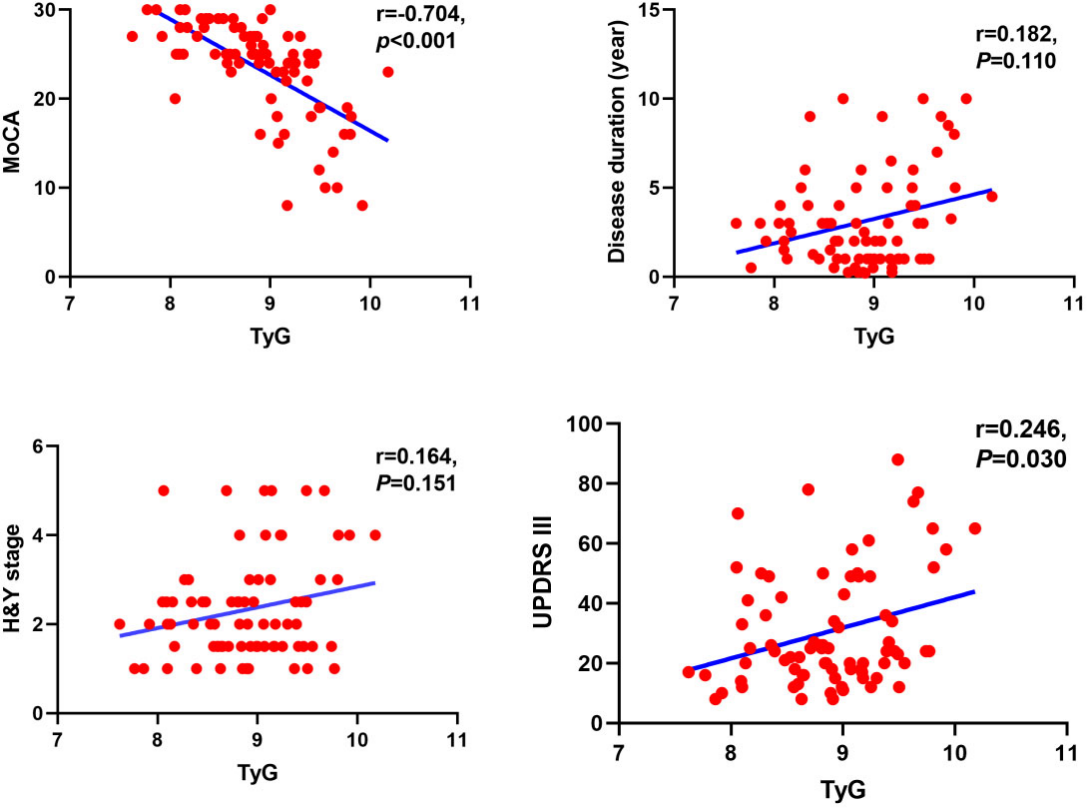
Correlation analysis of Parkinson’s disease duration and other related scale scores, including MoCA, H&Y stage, and UPDRS III, with TyG. The TyG was negatively correlated with MoCA (*r* = −0.704, *P* < 0.001), positively correlated with UPDRS III (*r* = 0.246, *p* = 0.030), but showed no significant correlation with disease duration (*r* = 0.182, *p* = 0.110) or H&Y stage (*r* = 0.164, *p* = 0.151).

**FIGURE 3:**
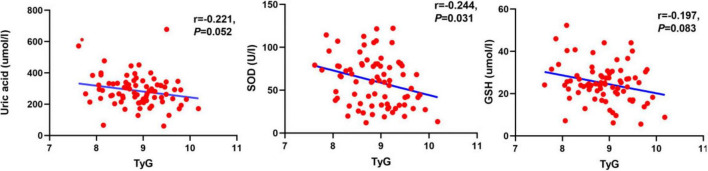
Analysis of the correlation between oxidative stress markers, including uric acid, SOD and SGH, and TyG. The TyG showed a significant negative correlation with SOD (*r* = −0.244, *p* = 0.031), while it showed a negative correlation trend with uric acid (*r* = −0.221, *p* = 0.052) and GSH (*r* = −0.197, *p* = 0.083), but the correlations were not significant.

### 3.4 Relationship between the TyG index and PDCI and PDD

Subsequently, multiple logistic regression analysis was used to explore the correlation between TyG and PDCI and PDD, with TyG as a continuous variable and tertile variable, respectively. The results showed that TyG was independently associated with PDCI, both as a continuous variable (OR = 6.177, 95% CI = 1.590–24.000) and as a tertile variable (OR = 5.478, 95% CI = 1.030–29.132), after adjusting for sex, age, education, H&Y, and duration of disease (Model 2 for PDCI). Nevertheless, for PDD, after adjusting for sex, age, education, H&Y, duration, UPDRS III, SOD, and GSH (Model 2 for PDD), TyG was independently associated with PDD only as a continuous variable, but not as a tertile variable (Tables 3, 4).

**TABLE 3:** Logistic regression analysis of the association of TyG index as a continuous variable with PDCI and PDD.

TyG index	PDCI		PDD	
Models	OR (95% CI)	*P*	OR (95% CI)	*P*
Unadjusted	11.881 (3.482, 0.538)	<0.001	17.575 (4.154, 4.366)	<0.001
Model 1	6.813 (1.907, 24.345)	0.003	12.276 (2.634, 7.216)	0.001
Model 2	6.177 (1.590, 24.000)	0.009	7.520 (1.147, 49.324)	0.036

triglyceride (TG) and fasting plasma glucose (FPG) were not included in the multivariable regression analysis because of collinearity. Model 1: adjusted for gender and age; Model 2: PDCI, adjusted for Model 1 + education level, H&Y, duration; PDD, adjusted for Model 1 + education level, UPDRS III, H&Y, duration, SOD, GSH. TyG, triglyceride-glucose; PDCI, Parkinson’s disease with cognitive impairment; PDD, dementia in Parkinson’s disease; OR, odds ratio; CI, confidence interval.

**TABLE 4:** Logistic regression analysis of the correlation between TyG index as tertile variable and PDCI and PDD.

TyG index	PDCI		PDD	
Models	OR (95% CI)	*P*	OR (95% CI)	*P*
**Unadjusted**				
Tertile 1	Reference		Reference	
Tertile 2	3.612 (1.147, 11.378)	0.028	4.861 (0.902, 26.193)	0.066
Tertile 3	13.033 (3.105, 54.704)	<0.001	17.045 (3.316, 87.607)	<0.001
**Model 1**				
Tertile 1	Reference		Reference	
Tertile 2	1.655 (0.447, 6.137)	0.451	2.363 (0.385, 14.520)	0.353
Tertile 3	6.867 (1.410, 33.444)	0.017	8.081 (1.408, 46.396)	0.019
**Model 2**				
Tertile 1	Reference		Reference	
Tertile 2	1.089 (0.247, 4.807)	0.911	0.637 (0.067, 6.093)	0.695
Tertile 3	5.478 (1.030, 29.132)	0.046	3.879 (0.547, 27.522)	0.175

triglyceride (TG) and fasting plasma glucose (FPG) were not included in the multivariable regression analysis because of collinearity. Model 1: adjusted for gender and age; Model 2: PDCI, adjusted for Model 1 + education level, H&Y, duration; PDD, adjusted for Model 1 + education level, UPDRS III, H&Y, duration, SOD, GSH. TyG, triglyceride-glucose; PDCI, Parkinson’s disease with cognitive impairment; PDD, dementia in Parkinson’s disease; OR, odds ratio; CI, confidence interval.

### 3.5 Correlation between the TyG index and PDCI in patients without diabetes mellitus

We again performed the logistic regression analysis in patients without DM to clarify whether glucose metabolic status could affect this association between TyG and PDCI. The results demonstrated that the independent correlation between TyG and PDCI still existed whether it was used as a continuous variable (OR = 5.478, 95% CI = 2.165–60.297, *P* = 0.004) or a tertile variable (OR = 10.819, 95% CI = 1.457–80.333, *P* = 0.002) (Table 5). The small diabetic subgroup (*n* = 9) precluded further stratified regression due to insufficient statistical power.

**TABLE 5:** Regression analysis of the correlation between TyG and PDCI in patients without diabetes mellitus.

TyG index	Unadjusted		Model 1		Model 2	
	OR (95% CI)	*P*	OR (95% CI)	*P*	OR (95% CI)	*P*
As continuous variable	16.667 (3.893, 71.348)	<0.001	10.858 (2.308, 1.070)	0.003	11.426 (2.165, 60.297)	0.004
**As tertile variable**						
Tertile 1	Reference		Reference			
Tertile 2	4.063 (1.216, 13.580)	0.023	1.857 (0.457, 7.541)	0.387	1.290 (0.251, 6.619)	0.760
Tertile 3	16.889 (3.179, 89.726)	<0.001	11.527 (1.767, 75.179)	0.011	10.819 (1.457, 80.333)	0.020

triglyceride (TG) and fasting plasma glucose (FPG) were not included in the multivariable regression analysis because of collinearity. Model 1: adjusted for gender and age; Model 2: PDCI, adjusted for Model 1 + education level, hypertension, UPDRS III, H&Y; PDD, adjusted for Model 1 + education level, hypertention, UPDRS III, H&Y, duration. TyG, triglyceride-glucose; PDCI, Parkinson’s disease with cognitive impairment; OR, odds ratio; CI, confidence interval.

### 3.6 Predictive value of TyG for PDCI

**FIGURE 4:**
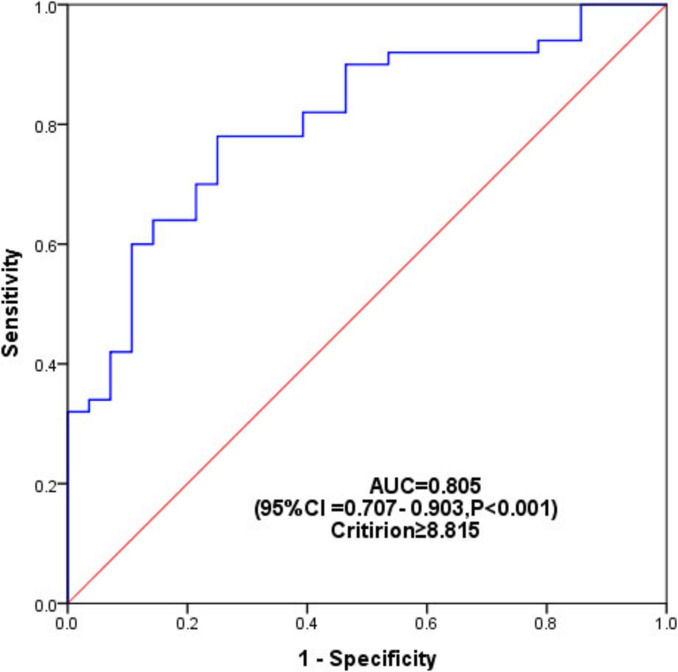
ROC analysis to evaluate the predictive value of TyG for PDCI. The area under the curve (AUC) was 0.805 (95% CI = 0.707 – 0.903, *P* < 0.001), and the optimal cut-off value for predicting PDCI by TyG was 8.815.

We further explored the predictive value of TyG for PDCI using ROC analysis, and the area under the curve (AUC) was 0.805 (95% CI = 0.707–0.903, *P* < 0.001) (Figure 4). The optimal cutoff value of TyG to predict PDCI was 8.815, and the sensitivity and specificity were 78% and 75%, respectively.

## 4 Discussion

This study provides epidemiological evidence to support the association between IR and PD, and it is the first to verify the independent association between the TyG index and PDCI. In this study, the proportion of PDCI was 65.4%, which was slightly higher than previous reports. This may be due to the fact that our center is a regional tertiary hospital, and all the subjects come from the inpatient department, where advanced and severe cases are overrepresented. According to our results, the TyG index was significantly higher in the PDCI subgroup than PDNC subgroups. Meanwhile, the TyG index was negatively correlated with the MoCA score and positively related to UPDRS III. TyG was independently associated with PDCI both as a continuous variable and as a tertile variable. Interestingly, we found that TyG was significantly associated with PDD when used as a continuous variable, but this independent association ceased to exist when used as a tertile variable. We speculate that this loss of significance in tertile based analyses may stem from reduced statistical power due to limited sample size. Considering the correlation between TyG and DM, we further performed the analysis in patients without DM. This correlation between the TyG index and PDCI still existed and seemed more robust. However, this trend needs to be further verified due to the limited sample size and small number of patients with DM. Meanwhile, we also found a correlation between TyG and UPDRS III, suggesting that TyG may also be related to PD motor symptoms. Since oxidative stress plays a vital role in both IR and PD, we further analyzed the correlation between TyG and peripheral blood oxidative stress markers, including uric acid, SOD, and GSH, and the results suggested that TyG and SOD were correlated. The result indicated that oxidative stress might be a potential mechanism for the correlation between the TyG index and PDCI. It should be noted that the statistical power for evaluating the observed effect (OR = 5.478) is 83% (calculated using the R package WebPower through Monte Carlo simulation), which exceeds the 80% threshold for conventional clinical studies. This indicates our study had sufficient power to identify this strong association. While the statistical power to detect the observed effect for PDD (OR = 3.879) was 48%, which explains non-significant *p*-value and wide CI.

Sandyk (1993) identified a link between T2DM and PD in 1993, highlighting that PD patients with T2DM tended to have more severe motor symptoms and weaker responses to treatment. They also found that 50%–80% of PD patients have abnormal glucose metabolism, although more recent studies have found that the proportion is about 20% (Athauda and Foltynie, 2016; Sandyk, 1993; Cheong et al., 2020). A study based on data from English National Hospital Episode Statistics suggested that the risk of developing PD was 1.3 times higher in people with diabetes than in those without (De Pablo-Fernandez et al., 2018). A recent large study of 1930 patients with PD indicated that PD patients with DM tend to have more severe motor and non-motor symptoms, as well as faster disease progression of PD (Athauda et al., 2022). IR has also been reported to be closely related to the severity, disease progression, and cognitive dysfunction of PD (Athauda and Foltynie, 2016; Peng et al., 2018; Hogg et al., 2018). Although the majority of studies support the adverse effects of DM and IR on PD, it should be noted that some studies have reported inconsistent results (Takamatsu et al., 2017; Molsberry et al., 2020). Therefore, their relationship still needs to be further studied.

The TyG index, as a relatively new surrogate index of insulin resistance, has been proven effective. Studies have shown that it is more stable, easily available, and cost-effective than other alternative markers, such as the Homeostatic Model Assessment of Insulin Resistance (HOMA-IR) and the quantitative insulin check index (QUICKI) (Wang et al., 2021). At present, it has been widely used in clinical practice. TyG has been confirmed by a large number of studies to be associated with the occurrence, severity, and poor prognosis of heart diseases, including coronary atherosclerotic disease and heart failure (Li et al., 2022; Jung et al., 2022; Liu et al., 2022). In addition, increasing studies have demonstrated the relationship between the TyG index and AD. According to a recent large cohort study, a high TyG index could significantly increase the risk of subsequent dementia, not only AD but also vascular dementia (Hong et al., 2021). In recent years, the potential correlation between TyG and stroke has been confirmed. Numerous studies have shown that TyG could escalate the risk of occurrence, recurrence, and poor prognosis of stroke (Wang et al., 2022; Yang et al., 2022; Wang et al., 2022). However, to our knowledge, the association between TyG and PD or PDCI has not been reported. Our study provides epidemiological evidence for their association.

The mechanism underlying the association between IR and PD remains unclear. However, growing evidence suggests that several mechanisms may play a role. First, impaired insulin signaling pathways, studies have found that insulin receptors in the periphery also exist in the brain (Kellar and Craft, 2020). Peripheral insulin can enter the brain through the blood-brain barrier to play an important neuroprotective role, such as transmitting dopamine, maintaining synapses, and regulating neuronal survival and growth (Burillo et al., 2021; Moran et al., 2019). IR involves impaired insulin signaling, which can extend to the brain and affect the neuroprotective function of insulin. Second, mitochondrial dysfunction, both IR and PD, is associated with impaired mitochondrial function. Insulin resistance leads to decreased mitochondrial biogenesis and increased oxidative stress, contributing to insufficient adenosine triphosphate production and increased neuronal vulnerability in PD (Butterfield et al., 2022; Ghowsi et al., 2021). The significant correlation between the TyG index and SOD in our study also confirmed the role of oxidative stress. Thirdly, dysregulation of insulin-degrading-enzyme (IDE), IDE is responsible for degrading insulin, the amyloid-β (Aβ) protein, and the α-synuclein. In the presence of IR, IDE may preferentially degrade insulin over Aβ and α-synuclein, resulting in abnormal deposition of Aβ and α-synuclein in the brain (Moran et al., 2019). Fourth, IR can lead to chronic low-grade inflammation, which is known to contribute to neurodegenerative processes. Inflammatory cytokines can cross the blood-brain barrier, promoting inflammation in the brain that may accelerate the loss of dopaminergic neurons (Peng et al., 2018; Cheong et al., 2020; Athauda et al., 2022). In addition, autophagy dysfunction has also been reported to play a role (Cullinane et al., 2023; Santiago et al., 2023).

Although this study demonstrated a potential correlation between the TyG index and PDCI, some limitations remain. First, while adequately powered for the primary PDCI analysis (83% power for OR = 5.478), the study was underpowered for PDD-specific outcomes (48% power for OR = 3.879). Moreover, due to the small sample size, particularly the relatively small number of diabetic patients, the impact of different glucose metabolism statuses on this result cannot be deeply explained. A larger cohort can provide a more in-depth and precise verification of the correlation between TyG and PDCI, especially in subgroup analyses. Second, a key limitation of this study was the lack of systematic data on medication usage, including dopaminergic therapy (e.g., levodopa equivalent daily dose), antidiabetics (e.g., metformin, insulin), and antihypertensives (e.g., angiotensin converting enzyme inhibitors, beta-blockers). These medications may influence metabolic conditions (including TyG levels) and cognitive function, potentially confounding the observed association. Given these complex interactions, future studies should rigorously document and adjust for medication usage data to clarify the true association between TyG and PDCI. Third, detailed cognitive subdomain assessment was not carried out with multiple cognitive scales, which could not be used further to evaluate the impact of TyG on cognitive subdomains. Finally, both TyG and cognition function were assessed only once, and the dynamic correlation of TyG on cognition function was unclear.

## 5 Conclusion

This study illustrated the independent correlation between the TyG index and PDCI. An elevated TyG index could be seen as a risk factor for PDCI, even in PD patients without DM. The underlying mechanism remains to be investigated in future studies.

## Data Availability

The raw data supporting the conclusions of this article will be made available by the authors, without undue reservation.

## References

[B1] Aarsland D. Creese B. Politis M. Chaudhuri K. R. Ffytche D. H. Weintraub D. (2017). Cognitive decline in Parkinson disease. *Nat. Rev. Neurol.* 13 217–231. 10.1038/nrneurol.2017.27 28257128 PMC5643027

[B2] Athauda D. Evans J. Wernick A. Virdi G. Choi M. L. Lawton M. (2022). The impact of type 2 diabetes in Parkinson’s disease. *Mov. Disord.* 37 1612–1623. 10.1002/mds.29122 35699244 PMC9543753

[B3] Athauda D. Foltynie T. (2016). Insulin resistance and Parkinson’s disease: A new target for disease modification? *Prog. Neurobiol.* 14 98–120. 10.1016/j.pneurobio.2016.10.001 27713036

[B4] Bastard J. P. Lavoie M. E. Messier V. Prud Homme D. Rabasa-Lhoret R. (2012). Evaluation of two new surrogate indices including parameters not using insulin to assess insulin sensitivity/resistance in non-diabetic postmenopausal women: A monet group study. *Diab. Metab.* 38 258–263. 10.1016/j.diabet.2012.01.004 22405724

[B5] Becker S. Granert O. Timmers M. Pilotto A. Van Nueten L. Roeben B. (2021). Association of hippocampal subfields, csf biomarkers, and cognition in patients with Parkinson disease without dementia. *Neurology* 96 e904–e915. 10.1212/WNL.0000000000011224 33219138

[B6] Burillo J. Marqués P. Jiménez B. González-Blanco C. Benito M. Guillén C. (2021). Insulin resistance and diabetes mellitus in Alzheimer’s disease. *Cells* 10:1236. 10.3390/cells10051236 34069890 PMC8157600

[B7] Butterfield D. A. Favia M. Spera I. Campanella A. Lanza M. Castegna A. (2022). Metabolic features of brain function with relevance to clinical features of Alzheimer and parkinson diseases. *Molecules* 27:951. 10.3390/molecules27030951 35164216 PMC8839962

[B8] Cheong J. L. Y. de Pablo-Fernandez E. Foltynie T. Noyce A. J. (2020). The association between type 2 diabetes mellitus and Parkinson’s disease. *J. Parkinsons Dis.* 10 775–789. 10.3233/JPD-191900 32333549 PMC7458510

[B9] Cullinane P. W. de Pablo Fernandez E. König A. Outeiro T. F. Jaunmuktane Z. Warner T. T. (2023). Type 2 diabetes and Parkinson’s disease: A focused review of current concepts. *Mov. Disord.* 38 162–177. 10.1002/mds.29298 36567671

[B10] de Pablo-Fernández E. Courtney R. Rockliffe A. Gentleman S. Holton J. L. Warner T. T. (2021). Faster disease progression in parkinson’s disease with type 2 diabetes is not associated with increased α−synuclein, tau, amyloid−β or vascular pathology. *Neuropathol. Appl. Neurobiol.* 47 1080–1091. 10.1111/nan.12728 33969516

[B11] De Pablo-Fernandez E. Goldacre R. Pakpoor J. Noyce A. J. Warner T. T. (2018). Association between diabetes and subsequent Parkinson disease: A record-linkage cohort study. *Neurology* 91 e139–e142. 10.1212/WNL.0000000000005771 29898968

[B12] Ghowsi M. Qalekhani F. Farzaei M. H. Mahmudii F. Yousofvand N. Joshi T. (2021). Inflammation, oxidative stress, insulin resistance, and hypertension as mediators for adverse effects of obesity on the brain: A review. *Biomedicine* 11 13–22. 10.37796/2211-8039.1174 35223415 PMC8823488

[B13] Hogg E. Athreya K. Basile C. Tan E. E. Kaminski J. Tagliati M. (2018). High prevalence of undiagnosed insulin resistance in non-diabetic subjects with Parkinson’s disease. *J. Parkinsons. Dis.* 8 259–265. 10.3233/JPD-181305 29614702

[B14] Hong S. Han K. Park C.-Y. (2021). The insulin resistance by triglyceride glucose index and risk for dementia: Population-based study. *Alzheimers Res. Ther.* 13:9. 10.1186/s13195-020-00758-4 33402193 PMC7786939

[B15] Hussein A. Guevara C. A. Del Valle P. Gupta S. Benson D. L. Huntley G. W. (2023). Non-motor symptoms of Parkinson’s disease: The neurobiology of early psychiatric and cognitive dysfunction. *Neuroscientist* 29 97–116. 10.1177/10738584211011979 33966533 PMC9338765

[B16] Jung M.-H. Yi S.-W. An S. J. Yi J.-J. Ihm S.-H. Han S. (2022). Associations between the triglyceride-glucose index and cardiovascular disease in over 150,000 cancer survivors: A population-based cohort study. *Cardiovasc. Diabetol.* 21:52. 10.1186/s12933-022-01490-z 35429972 PMC9013459

[B17] Kellar D. Craft S. (2020). Brain insulin resistance in Alzheimer’s disease and related disorders: Mechanisms and therapeutic approaches. *Lancet Neurol.* 19 758–766. 10.1016/S1474-4422(20)30231-3 32730766 PMC9661919

[B18] Li H. Zuo Y. Qian F. Chen S. Tian X. Wang P. (2022). Triglyceride-glucose index variability and incident cardiovascular disease: A prospective cohort study. *Cardiovasc. Diabetol.* 21:105. 10.1186/s12933-022-01541-5 35689232 PMC9188105

[B19] Litvan I. Goldman J. G. Tröster A. I. Schmand B. A. Weintraub D. Petersen R. C. (2012). Diagnostic criteria for mild cognitive impairment in Parkinson’s disease: Movement disorder society task force guidelines. *Mov. Disord.* 27 349–356. 10.1002/mds.24893 22275317 PMC3641655

[B20] Liu X. Tan Z. Huang Y. Zhao H. Liu M. Yu P. (2022). Relationship between the triglyceride-glucose index and risk of cardiovascular diseases and mortality in the general population: A systematic review and meta-analysis. *Cardiovasc. Diabetol.* 21:124. 10.1186/s12933-022-01546-0 35778731 PMC9250255

[B21] Molsberry S. Bjornevik K. Hughes K. C. Zhang Z. J. Jeanfavre S. Clish C. (2020). Plasma metabolomic markers of insulin resistance and diabetes and rate of incident Parkinson’s disease. *J. Parkinsons Dis.* 10 1011–1021. 10.3233/JPD-191896 32250318 PMC8034435

[B22] Moran C. Beare R. Wang W. Callisaya M. Srikanth V. Alzheimer’s Disease Neuroimaging Initiative [ADNI] (2019). Type 2 diabetes mellitus, brain atrophy, and cognitive decline. *Neurology* 92 e823–e830. 10.1212/WNL.0000000000006955 30674592 PMC7987953

[B23] Peng Z. Dong S. Tao Y. Huo Y. Zhou Z. Huang W. (2018). Metabolic syndrome contributes to cognitive impairment in patients with Parkinson’s disease. *Parkinson. Relat. Disord.* 55 68–74. 10.1016/j.parkreldis.2018.05.013 29908727

[B24] Sandyk R. (1993). The relationship between diabetes mellitus and Parkinson’s disease. *Int. J. Neurosci.* 69 125–130. 10.3109/00207459309003322 8082998

[B25] Santiago J. A. Karthikeyan M. Lackey M. Villavicencio D. Potashkin J. A. (2023). Diabetes: A tipping point in neurodegenerative diseases. *Trends Mol. Med.* 29 1029–1044. 10.1016/j.molmed.2023.09.005 37827904 PMC10844978

[B26] Simental-Mendía L. E. Rodríguez-Morán M. Guerrero-Romero F. (2008). The product of fasting glucose and triglycerides as surrogate for identifying insulin resistance in apparently healthy subjects. *Metab. Syndr. Relat. Disord.* 6, 299–304. 10.1089/met.2008.0034 19067533

[B27] Takamatsu Y. Ho G. Koike W. Sugama S. Takenouchi T. Waragai M. (2017). Combined immunotherapy with “anti-insulin resistance” therapy as a novel therapeutic strategy against neurodegenerative diseases. *NPJ Parkinsons Dis.* 3:4. 10.1038/s41531-016-0001-1 28649604 PMC5445606

[B28] Wang S. Shi J. Peng Y. Fang Q. Mu Q. Gu W. (2021). Stronger association of triglyceride glucose index than the HOMA-IR with arterial stiffness in patients with type 2 diabetes: A real-world single-centre study. *Cardiovasc. Diabetol.* 20:82. 10.1186/s12933-021-01274-x 33888131 PMC8063289

[B29] Wang X. Feng B. Huang Z. Cai Z. Yu X. Chen Z. (2022). Relationship of cumulative exposure to the triglyceride-glucose index with ischemic stroke: A 9-year prospective study in the Kailuan cohort. *Cardiovasc. Diabetol.* 21:66. 10.1186/s12933-022-01510-y 35505313 PMC9066788

[B30] Yang X. Wang G. Jing J. Wang A. Zhang X. Jia Q. (2022). Association of triglyceride-glucose index and stroke recurrence among nondiabetic patients with acute ischemic stroke. *BMC Neurol.* 22:79. 10.1186/s12883-022-02588-3 35260102 PMC8902785

